# Alterations in microbiota and their metabolites are associated with beneficial effects of bile acid sequestrant on icteric primary biliary Cholangitis

**DOI:** 10.1080/19490976.2021.1946366

**Published:** 2021-08-26

**Authors:** Bo Li, Jun Zhang, Yong Chen, Qixia Wang, Li Yan, Rui Wang, Yiran Wei, Zhengrui You, Yikang Li, Qi Miao, Xiao Xiao, Min Lian, Weihua Chen, Dekai Qiu, Jingyuan Fang, M. Eric Gershwin, Ruqi Tang, Xiong Ma

**Affiliations:** a Division of Gastroenterology and Hepatology, Key Laboratory of Gastroenterology and Hepatology, Ministry of Health, State Key Laboratory for Oncogenes and Related Genes, Renji Hospital, School of Medicine, Shanghai JiaoTong University; Shanghai Institute of Digestive Disease;145 Middle Shandong Road, Shanghai, China; b Division of Rheumatology, Department of Medicine, Allergy and Clinical Immunology, University of California at Davis, Davis, CA, USA

**Keywords:** Microbiome, bile acids, cholestasis, metabolome, short-chain fatty acids

## Abstract

**Background**: Increasing data suggests an interaction between bile acids and intestinal microbiota in the pathogenesis of primary biliary cholangitis (PBC). Bile acid sequestrants are widely used to bind bile acids in the intestinal lumen and are therefore posited to impact gut bacteria. Herein we aimed to investigate the effects of cholestyramine on the bile acid profile and gut microbiome in a cohort of icteric PBC patients.

**Results:** Thirty-three PBC patients were treated with cholestyramine, serum and stool samples were collected at baseline, 4 and 16 weeks. Shotgun metagenomic sequencing and targeted metabolomic profiling were performed. Following cholestyramine administration, patients exhibited a high interpersonal variability in remission of cholestasis, and were therefore dichotomized according to the decrease of total bilirubin. Gut microbial co-abundance networks showed distinct taxa interactions between subjects with superior remission (SR) and those with inferior remission (IR) at baseline. After treatment, compositional shifts of the microbiome in the SR group were characterized with enrichment of two *Lachnospiraceae* species, typically producing short-chain fatty acids (SCFAs). In contrast, *Klebsiella pneumonia*, a commensal pathobiont, was only increased in the IR group. Correspondingly, metabolome analysis demonstrated that patients with SR, but not IR, were marked by elevations of SCFAs including valeric acid and caproic acid. Finally, integrative analysis identified robust associations between the variations of microbiota, metabolites, and inflammatory cytokines in SR group, indicating potential mechanistic links.

**Conclusions:** Beneficial responses caused by cholestyramine were closely related with compositional and functional alterations in gut commensal, highlighting the possibility of exploring bile acid-microbiota interactions for treating PBC.

## Introduction

Primary biliary cholangitis (PBC) is an autoimmune liver disease characterized by the presence of antimitochondrial antibodies (AMA) and progressive destruction of interlobular bile ducts.^1^ Ursodeoxycholic acid (UDCA) is effective in approximately two-thirds of early-stage PBC patients and improves life expectancy without additional therapies.^2,3^ Emerging evidence suggests a critical role for the excessive toxic bile acids and gut dysbiosis in the pathogenesis of PBC.^1,3^

Interactions between bile acids and the gut microbiome have been well established. By acting on the farnesoid X receptor (FXR) and Takeda G protein-coupled receptor 5 (TGR5), bile acids are involved in multiple signaling pathways, including metabolism, fibrosis, and immune homeostasis.^4,5^ Gut microbiota metabolize bile acids with defined enzymes, and thereby impact the bile acid signalings.^4^ For example, supplementation of *Lactobacillus rhamnosus* prevented liver fibrosis in a mouse model of cholestasis through upregulating intestinal FXR signaling.^6^ In turn, dynamics of bile acids also exert a profound impact on the intestinal microbiome. The use of a bile acid analog, obeticholic acid (OCA), led to a reversible induction of gram-positive bacteria in rodents and humans.^7^ Of note, our previous work revealed the presence of a microbial perturbation in naïve PBC patients, and UDCA treatment can partially reverse this dysbiosis.^8,9^ In addition, levels of secondary bile acids were inversely correlated with PBC-enriched gut bacteria.^10^ Nevertheless, how alterations of bile acids modulate gut microbiota in PBC remains elusive.

Cholestyramine is one of the bile acid sequestrants capable of binding to intestinal lumen bile acids and has been used to treat cholestatic pruritus with a good safety profile and accessibility.^11^ In the Mdr2 knockout mouse model, bile acid sequestrants alleviate cholestatic liver and bile duct injury^12^ and, in humans with primary sclerosing cholangitis (PSC), there are case reports of improvement of cholestasis by cholestyramine.^13^ Here, we performed a 16-week longitudinal study in icteric PBC subjects using cholestyramine to characterize the compositional and functional responses of gut microbiota to alterations in endogenous bile acid levels. Multi-omic analysis including shotgun metagenomic sequencing and targeted metabolomic profiling was utilized. We further investigated whether these changes in bile acids and microbiota could explain the beneficial effects of cholestyramine on cholestasis.

## Materials and methods

### Study subjects

All subjects were enrolled from the outpatient clinical of Renji Hospital, affiliated to Shanghai Jiao Tong University School of Medicine between January 2017 and ending March 2018. The mean age of the subjects was 48.8 years (SD 9.1), and 27/33 (81.8%) were women. All the patients took a standard dose of 13–15 mg/kg/d UDCA at baseline and throughout the study. The mean baseline total bilirubin level was 95.09 μmol/L (Supplementary Table 1).

Patients were enrolled on a consecutive basis if eligible. All patients were adults with a confirmed diagnosis of PBC and severe cholestasis and all had been treated with standard UDCA therapy for at least 6 months. Exclusion criteria included malignancy, renal dysfunction, pregnancy, or lactation. None of the patients had previously received cholestyramine. Patients were administered twice-daily cholestyramine at a dose of 8 g each time prior to meals, with a 4-hour window before administration of other medications, to avoid drug interference in intestinal absorption of UDCA. Liver biochemistries were performed at baseline and weeks 4 and 16. Serum and stool samples were collected on each visit. Thirty-three patients received cholestyramine treatment for 4 weeks, 28/33 received cholestyramine for 16 weeks, and 5/33 patients discontinued the study voluntarily after the 4-week sample period because they were unable to tolerate the “taste” of the cholestyramine. Patients enrolled did not take any antibiotics, PPI or metformin throughout the intervention. None has had encephalopathy, cholangitis or gastrointestinal hemorrhage during this period.

Written informed consent was obtained from all patients and the study was conducted in accordance with the principles of the Declaration of Helsinki and was approved by the Ethics Committee of Renji Hospital, School of Medicine, Shanghai Jiao Tong University (#2013-030).

## Stratification of the subjects

For further analysis, patients were stratified into two groups according to changes of total bilirubin following 16-week treatment of cholestyramine. Subjects with a delta percent (Δ%) of total bilirubin higher than the median were allocated to the group with superior remission (SR), while subjects with Δ% of total bilirubin lower than the median were assigned to the inferior remission (IR) group.

## Sample preparation

Patients were required to fast overnight before collection of blood samples on the morning of each visit. Blood samples were centrifuged at 2800 rpm for 15 minutes at 4°C and serum were aliquoted and stored at −80°C until analysis. Fecal samples were freshly collected and immediately frozen at −80°C.

### UPLS-MS/MS measurement of bile acids and C4

Bile acids and C4 were quantified by ultra-performance liquid chromatography coupled to tandem mass spectrometry (UPLC-MS/MS) system (ACQUITY UPLC-Xevo TQ-S, Waters Corp., Milford, MA, USA).^14,15^ Briefly, 180 μL of acetonitrile/methanol (8:2) containing internal standards was added to 20 μL of serum samples in a 96-well plate. Internal standard concentrations were kept constant at all the calibration points (150 nM for GCA-d4, TCA-d4, TCDCA-d9, UDCA-d4, CA-d4, GCDCA-d4, GDCA-d4, DCA-d4, LA-d4, and β-CA-d5). The mixture was then vortexed at 1500 rpm for 2 min at 10°C and centrifuged at 13000 rpm for 20 min at 4°C. The supernatant was transferred to another plate and vacuum-dried. The residues were reconstituted with equal volume of acetonitrile/methanol (8:2) and water, and then centrifuged at 13000 rpm for 20 min at 4°C. After centrifugation, the supernatant (5 μL) was injected into the system for analysis. UPLC-MS/MS raw data were obtained and processed using MassLynx 4.1 software (Waters Corp., Milford, MA, USA).

### UPLS-MS/MS profiling of serum microbiota-derived metabolites

Quantification of the microbial metabolites was performed by Metabo-profile (Shanghai, China) using a UPLC-MS/MS system. All targeted standards were obtained from Sigma–Aldrich (St. Louis, MO, USA). The serum samples were processed as previously described.^16^ Briefly, 25 μL serum samples were extracted with 100 μL cold methanol by centrifuging at 4000 g for 30 min. Then 30 μL supernatant underwent derivatization at 30°C for 60 min and was subsequently diluted with cold 50% methanol and stored at −20°C for 20 min. After that, the mixture was centrifuged at 4000 g for 30 min, and 135 μL of supernatant was transferred to a new 96-well plate with 15 μL internal standards and finally subjected to LC-MS analysis. To ensure reproducibility, the quality control samples were prepared with the test samples and injected at every 14 test samples throughout the process.

### DNA extraction and metagenomic sequencing

Genomic DNA was extracted from feces using QIAamp PowerFecal DNA Kit (Qiagen, USA). DNA concentration was quantitated using a Quantus Fluorometer (Promega, CA, USA) and quality checked on a 1% agarose gels electrophoresis system. Whole-genome shotgun sequencing libraries were prepared using a TruSeq^TM^ DNA Sample Prep Kit (Illumina, San Diego, CA, USA). Individual libraries were pooled and then sequenced on a HiSeq 4000 platform (Illumina, CA, USA), using a 150-bp paired-end read protocol.

### Metagenomic sequencing data processing

Raw sequencing reads were first quality filtered using KneadData (version 0.7.2). Briefly, low-quality reads were trimmed with Trimmomatic, setting the minimum length to 50% of the total input read length. Human DNA reads were removed with Bowtie 2. Taxonomic profiles of quality-filtered metagenomes were generated using MetaPhlAn2 (version 2.7.7) with default parameters.^17^ In our study, species-level data were considered and reported as relative abundance. Only species with relative abundance higher than 0.001% and present in at least 20% of the total samples were kept for further analysis. Functional profiling was performed using HUMAnN2 (version 0.11.2) and summarized as KEGG (Kyoto Encyclopedia of Genes and Genomes Statistical analysis) pathways, which were normalized to counts per million (CPM).^18^ Pathways present in less than 20% of the samples were not included in the analysis.

### Measurement of serum FGF19 and GLP-1

Serum levels of FGF19 and active GLP-1 were assayed using FGF19 Quantikine ELISA kit (R&D Systems, USA) and GLP-1(7–36) *in vitro* SimpleStep ELISA ® kit (Abcam, UK), respectively.

### Measurement of serum inflammatory cytokines

Serum levels of IL-1β, IFN-γ, TNF-α, MCP-1, IL-6, IL-8, IL-12p70, IL-17A, IL-18, IL-23 were measured by a LEGENDplex^TM^ Human Inflammation Panel 1 (Biolegend, USA).

## Statistical analysis

Statistical analyses were performed using R packages (version 3.5.2) or SPSS (version 24). Alpha-diversity was reported as Shannon index, using diversity function from vegan package. Beta-diversity was based on Bray–Curtis dissimilarities of taxonomic species assessed by vegdist function from the vegan package. A two-tailed Wilcoxon signed-rank test was used to analyze paired groups for bile acids, microbial metabolites and clinical data. Longitudinal generalized linear mixed models (GLMMs) were constructed for metagenomic taxa and cytokines using the lmer function from package lmerTest. Time (week) was set as fixed effect and subject as a random effect in the longitudinal GLMMs. The relative abundances of species were centered log ratio transformed before analysis. For correlation analysis, we applied repeated measures correlation (rmcorr) methods to test for associations between variables (bile acids, microbial taxa, metabolites and cytokines) within each subject. The Benjamini–Hochberg method was used to adjust *p* values for multiple testing.

## Co-abundance network analysis

We constructed microbial co-abundance network for samples at baseline and after 16-weeks of cholestyramine, respectively. SparCC was applied to compute the microbial correlations between species with relative abundance higher than 0.001% and present in at least 20% of the samples.^19^ The correlation values larger than 0.3 or smaller than −0.3 with *p* value <.05 were retained for network visualization in Cytoscape.^20^

## Results

### Variability in biochemical responses to the bile acid sequestrant

**Figure 1.**
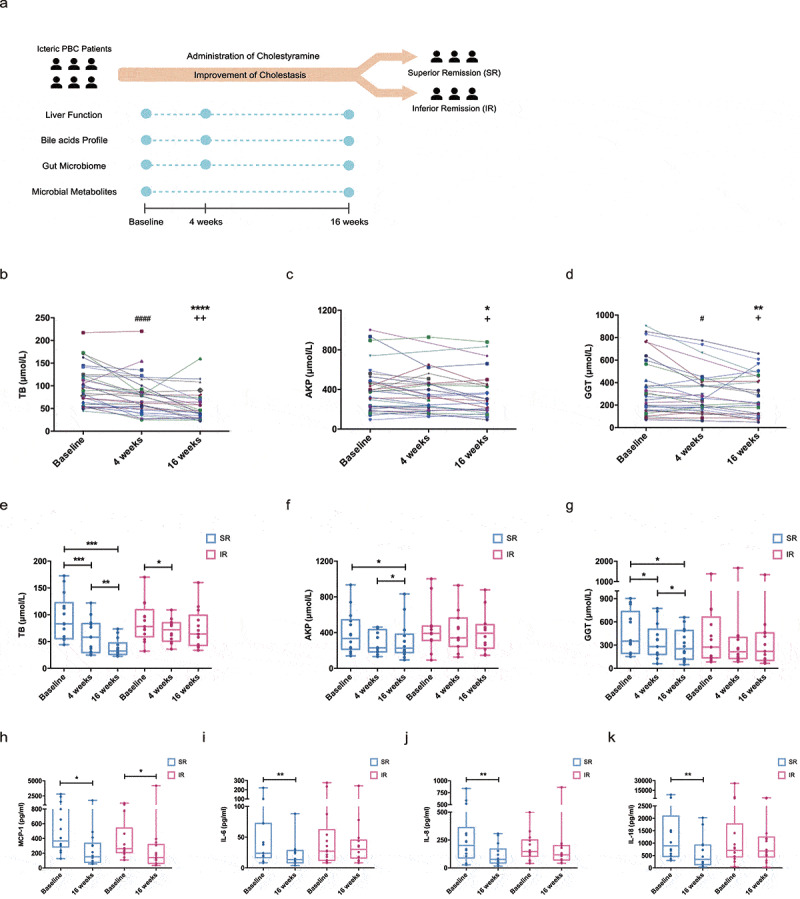
**Variability in biochemichal responses to cholestyramine**. (a) An outline of the study. Linear plots depict changes in (b) total bilirubin levels, (c) ALP levels and (d) GGT levels of individual subjects in response to 4- and 16-week treatment of cholestyramine. Paired Wilcoxon rank-sum test was used. * *p* < .05, ** *p* < .01, **** *p* < .0001: Baseline *vs* 16 weeks. ^#^ *p* < .05, ^####^ *p* < .0001: Baseline *vs* 4 weeks. ^+^ *p* < .05: 4 weeks *vs* 16 weeks. Patients were further stratified according to the decrease of bilirubin. Changes of (e) total bilirubin, (f) ALP, (g) GGT in group with superior remission (SR) and inferior remission (IR), respectively. Serum levels of (h) MCP-1, (i) IL-6, (j) IL-8 and (k) IL-18 in patients of two subgroups.* *p* < .05, ** *p* < .01, *** *p* < .001. ALP, alkaline phosphatase; GGT, γ-glutamyl transpeptidase. MCP-1, monocyte chemoattractant protein-1; IL-6, interleukin-6; IL-8, interleukin-8; IL-18, interleukin-18

Patients were administered cholestyramine for 16 weeks and provided serum and fecal samples at the timepoints of week 0, 4, 16 (Figure 1a). A significant reduction of total bilirubin was observed at 4 weeks (paired Wilcoxon rank-sum test, *p* < .001, 4 weeks *vs* baseline; Figure 1b) and the bilirubin level further declined during follow-up visits (paired Wilcoxon rank-sum test, *p* < .0001,16 weeks *vs* Baseline; p < .01, 16 weeks *vs* 4 weeks; Figure 1b). In parallel, levels of alkaline phosphatase (ALP) and γ-glutamyl transpeptidase (GGT) declined compared with baseline, to a modest but significant extent (Figure 1c, d,). Other details of liver biochemistry changes are shown in Supplementary Table 1.

We observed a high interpersonal variability in the amelioration of hyperbilirubinemia, which suggested a heterogeneous response to the treatment of cholestyramine. As such, subjects were further stratified according to the median decrease of bilirubin at 16 weeks, i.e., group with superior remission of cholestasis (SR, n = 14) and group with inferior remission (IR, n = 14). The baseline comparison of these two groups is shown in Table 1. There was no difference in the age, sex and BMI between the two subgroups. Consistent with a lower bilirubin in patients with SR and cholestasis parameters, ALP and GGT were lower as well (Figure 1e, f, g). A concordant decrease of peripheral inflammatory cytokines was observed in patients in SR group following treatment of bile acid sequestrant (Figure 1(h, i, j, k). These cytokines, including monocyte chemoattractant protein-1 (MCP-1), interleukin-6 (IL-6), interleukin-8 (IL-8) and interleukin-18 (IL-18), are overexpressed in PBC and contribute to the reactive phenotype of cholangiocyte.^21^

### Bile acid profile and canonical bile acid signaling were altered

**Figure 2.**
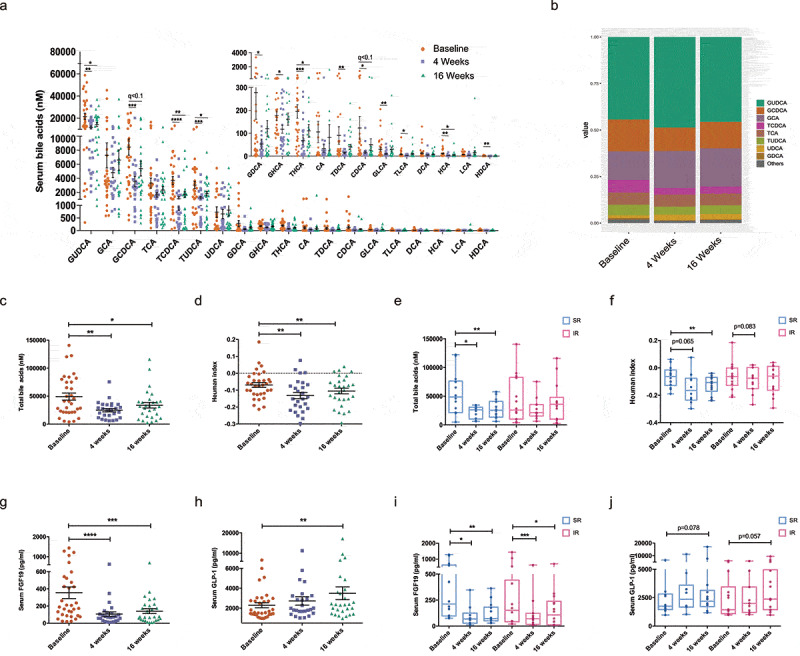
**Effects of cholestyramine on serum bile acid profile and intestinal bile acid signaling**. (a)Dot plots (with mean±SEM) showed the dynamics of 19 serum bile acids at 0, 4 and 16 weeks of cholestyramine treatment in all subjects. A paired Wilcoxon rank-sum test was used. * q < 0.05, ** q < 0.01, *** q < 0.001, **** q < 0.0001. (b) Composition of the serum bile acids at 0, 4 and 16 weeks of cholestyramine treatment. Changes of (c) total bile acids and (d) the Heuman index of bile acids before and after the 4- and 16-week treatment of cholestyramine. Decrease of Heuman index suggested less hydrophobicity of bile acids. (e) Total bile acids and (f) the Heuman index were analyzd within group SR and IR, respectively. * *p* < .05, ** *p* < .01. (f, g) Changes of serum FGF19 and active GLP-1 in all the subjects. ** *p* < .01, *** *p* < .001, **** *p* < .0001. (h, i) Separate analysis of levels of FGF19 and GLP-1 in group SR and IR. * *p* < .05, ** *p* < .01, *** *p* < .001. FGF19, fibroblast growth factor 19; GLP-1, glucagon-like peptide-1

Overall, treatment of cholestyramine markedly reduced circulating levels of bile acid and altered its composition (Figure 2(a, b)). The total bile acids decreased significantly (Figure 2c). To measure the polarity of the bile acid pool, hydrophobicity index of bile acids was calculated using Heuman’s algorithm.^22^ As a result, the hydrophobicity index of circulating bile acids declined following cholestyramine treatment (Figure 2d, paired Wilcoxon rank-sum test, *p* < .01, 4 weeks *vs* Baseline; *p* < .01, 16 weeks *vs* Baseline), possibly due to the prior sequestration of hydrophobic bile acids by cholestyramine. Of note, the reduction of serum total bile acids, as well as the decrease of bile acid hydrophobicity, were more prominent in group SR than IR (Figure 2(e, f)). In addition to shifting the polarity of the circulating bile acid pool, cholestyramine treatment also lowered the ratio of taurine/glycine bile acid in two groups (Supplementary SFigure 1a, b). The serum ratios of unconjugated/conjugated bile acid or secondary/primary bile acid were essentially unchanged (data not shown).

With regard to the fecal bile acid profile, cholestyramine facilitated the excretion of bile acids in feces (Supplementary SFigure 2a-c). The proportions of secondary bile acids,in particular, DCA and LCA, increased substantially (Supplementary SFigure 2b). The level of unconjugated bile acids increased, while the conjugated bile acids were not different (Supplementary SFigure 2d-g). The hydrophobicity index of fecal bile acids exhibited a tendency of increase (*p* = .095, Supplementary SFigure 2h). Notably, changes of fecal bile acids were more prominent in SR group, comparing with IR group (Supplementary SFigure 2i, j).

Next, canonical signaling of bile acid was examined. The level of FGF19 reduced profoundly following the bile acid sequestrant treatment in all the subjects (paired Wilcoxon rank-sum test, *p* < .0001, 4 weeks *vs* baseline; *p* < .001, 16 weeks *vs* baseline, figure 2f). A reduction in circulating FGF19 level indicates an impaired activation of FXR signaling in the ileal and thereby less suppression of CYP7A1, the key rate-limiting enzyme of bile acid synthesis. However, we measured the serum level of C4, a well-established marker of bile acid synthesis in liver, and found no significant changes after the 16-week treatment of cholestyramine (Supplementary SFigure 1b). In contrast to FGF19, we observed an increase in the secretion of active glucagon-like peptide-1 (GLP-1) (paired Wilcoxon rank-sum test, *p* < .01, 16 weeks *vs* baseline, Figure 2g). GLP-1 is one of the two identified incretins maintaining glucose homeostasis and is likely produced by enteroendocrine L cells residing in the distal intestine. Activation of TGR5 on enteroendocrine L cell triggers the release of GLP-1.^23^ In line with that, an increase of GLP-1 has also been observed in Mdr2^−/-^ mice treated with bile acid sequenstrant and conferred the cholangioprotective effects.^12^ Indeed, GLP-1 and its analog extendin-4 were proposed to prevent cholangiocyte from apoptosis and facilitate its proliferative repair in response to cholestasis.^24,25^ Patients in the two subgroups exhibited similar changes in FXR/FGF19 and TGR5/GLP-1 signaling in response to cholestyramine (Figure 2h j), suggesting a comparable alteration of bile acid signaling.

### Bile acid sequestrant modulated gut microbiota

**Figure 3.**
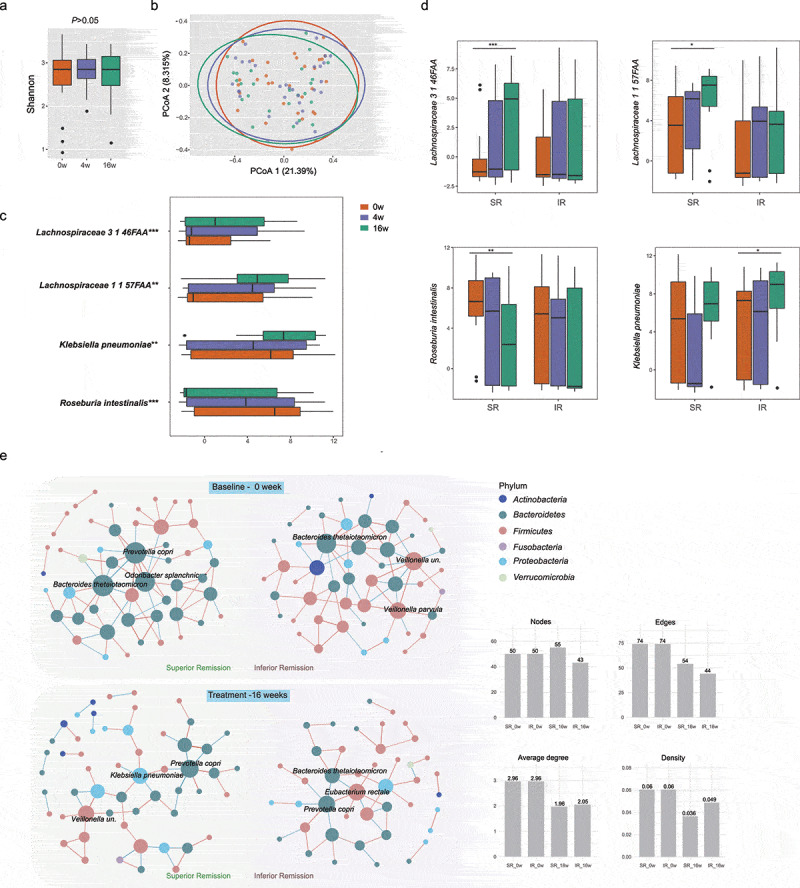
**Modulation of gut microbiota composition by cholestyramine in PBC**. (a-c) Alterations of gut microbiome in all the subjects in response to cholestyramine treatment. (a) Alpha diversity (shannon index) was measured at baseline, 4 weeks and 16 weeks using longitudinal GLMMs. (b) Principal coordinate analysis (PCoA) of species based on Bray–Curtis dissimilarities did not reveal shifts of the overall microbial compositions after 4 weeks or 16 weeks of treatment. (c) The relative abundance of four species significantly changed in response to cholestyramine intervention (longitudinal GLMMs, fdr<0.2). (d, e) Subjects were further classified into superior remission (SR) group and inferior remission (IR) group. (d) The species showed different alterations in response to treatment in SR and IR. (e) Microbial co-abundance analysis at species level of SR and IR was visualized in Cytoscape at baseline and 16 weeks of treatment. Strong correlations (|*r*| value > 0.3 and *P* < .05) are depicted. The red and blue edges denote positive and negative correlations, respectively. The color of the nodes is based on phylum and the size is based on edges connected to the nodes. Boxes represent the 25th–75th percentile of the distribution; the median is shown as a thick line in the middle of the box; whiskers extend to values with 1.5 times the difference between the 25th and 75th percentiles; and outliers are represented as dots. *p < .05, ** *p* < .01, *** *p* < .001

We performed whole-genome shotgun sequencing on fecal samples and obtained an average of 25.5 million paired-end reads per sample (min: 19.6 million, max: 30.7 million) after quality filtering. A longitudinal generalized linear mixed model (GLMM) was constructed for microbiome analysis. First, alpha diversity did not alter in response to the treatment of cholestyramine (Figure 3a). Principal coordinate analysis (PCoA) of Bray–Curtis dissimilarity was used to evaluate the global compositional shift of the gut microbiome. Compared to the baseline, there were no overall alterations in the microbial composition after the intervention (PERMANOVA, *p* > .05; Figure 3b), possibly due to the similar abundance of the most abundant species among these groups (Supplementary Figure 3). Nevertheless, four species suggested time-dependent alterations in response to cholestyramine, using longitudinal generalized linear mixed models (GLMMs, fdr<0.2, Figure 3c, Supplementary table 2). These included an increase in two *Lachnospiraceae* species (3146FAA and 1157FAA), known to be involved in production of short-chain fatty acids (SCFA), as well as a decrease in *Roseburia intestinalis*, which was reported to express mimotopes and trigger autoimmunity via cross-reactivity. Interestingly, we found a negative correlation between the changes of total bilirubin and these two *Lachnospiraceae* species in our PBC cohort (Supplementary SFigure 4). Surprisingly, the abundance of *Klebsiella pneumoniae*, found increased in UDCA-naïve PBC in our previous study, significantly increased following cholestyramine treatment. We also examined functional changes in the gut microbiome. However, no pathways achieved statistical significance after correction for multiple comparisons (data not shown).

### Divergent alterations of gut microbiota between patients with superior remission (SR) and inferior remission (IR)

Given the high inter-individual variability in the gut microbial taxa, we sought to determine whether the divergent responses to cholestyramine were associated with differential alterations in the gut microbiome. No obvious difference in alpha or beta diversity before and after intervention was found in patients of group SR or IR (Supplementary SFigure 5). At the taxonomic level, the species *Lachnospiraceae 3146FAA*, which demonstrated the greatest alteration in response to cholestyramine in the whole group, increased in patients of SR group (GLMMs, *P* = .00039), whereas it remained unchanged in IR group (Figure 3d). Likewise, the alterations of *Lachnospiraceae 1157FAA* and *Roseburia intestinalis* were detected only in SR group (*P* < .05, Figure 3d). On the contrary, the significant increase of *Klebsiella pneumoniae* found in all the participants occurred in IR group (*P* < .05, Figure 3d), but not SR group. In line, there was substantially more connections between variations of fecal bile acids and species in SR group, compared with IR. Enrichment of the *Lachnospiraceae* family correlated positively with the upregulated secondary bile acids (DCA and LCA) in SR group, as well as the hydrophobicity index of bile acids (Supplementary figure 6).

### Gut microbial co-abundance network suggested difference between SR and IR groups

We next performed co-abundance network analysis using SPARCC to interrogate the microbial relationships in the cohort (Figure 3e). Interestingly, very few co-abundance associations were found in the ecosystem of all the samples before and after treatment (Supplementary SFigure 7), but stratifying the participants into two subgroups resulted in considerably more complex networks (Figure 3e). More importantly, distinct patterns of microbial interactions existed between the SR and IR groups. At baseline, the connectivity in SR group was dominated by taxa in the phylum *Bacteriodetes*, whereas enhanced associations between taxa in *Firmicutes* were present in IR group. After intervention, the density of the networks decreased in both groups with a lower average degree, while SR group appeared to have more nodes and connections in comparison with IR group. Additionally, the species with the highest degree centrality in the networks, including *Bacteroides thetaiotaomicron* and *Prevotella copri*, were found to be differentially altered in response to the cholestyramine between the two subgroups (Supplementary SFigure 8). *P. copri*, associated with several chronic inflammatory diseases, such as rheumatoid arthritis, was increased only in the IR group. Taken together, these findings indicate that the discrepancies in species co-abundances may underlie the variability of responsiveness to the resin.

### Distinct changes of microbial metabolites in SR and IR groups

**Figure 4.**
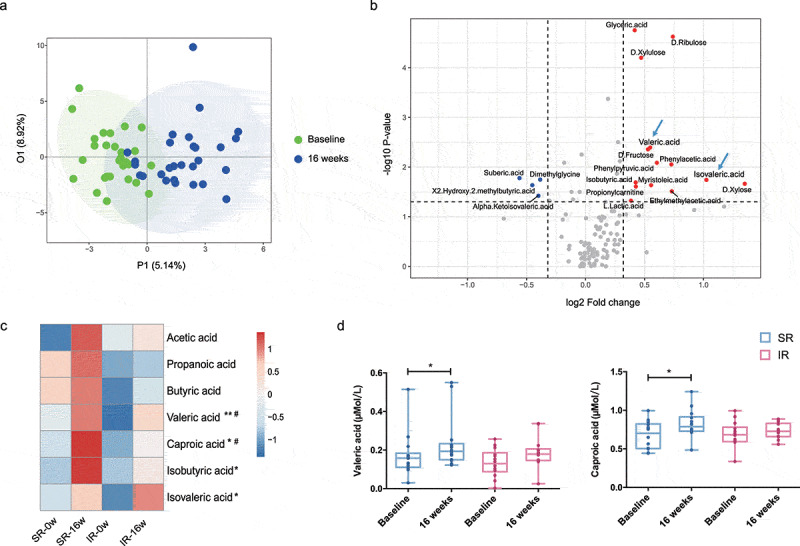
**Distinct changes of microbial metabolites between SR and IR groups**. (a) OPLA-DA score plots depicted the shift of circulating bacterial metabolites before and after the 16-week treatment of cholestyramine (R^2^Y = 0.65, Q^2^Y = 0.165). (b) Volcano plots showed individual metabolite altered in response to the intervention (*p* < .05). Dots in red and blue denote the up-regulated and down-regulated metabolites, respectively. (c) Heatmap showed the median fold changes of short-chain fatty acids (SCFAs) concentrations in SR and IR groups before and after the 16-week treatment. ^#^ *p* < .05 by paired Wilcoxon rank-sum test within group SR. * *p* < .05, ** *p* < .01 by paired Wilcoxon rank-sum test in all subjects. (d,e) Differential changes of valeric acid and caproic acid in SR and IR groups. * *p* < .05 by paired Wilcoxon rank-sum test within group SR or IR

The gut microbiota constantly produces large amounts of metabolites, which can enter the circulation and act as important signaling molecules at the extraintestinal organs. To further understand how bacteria impact host physiology, we performed microbiota-related metabolomics analysis of serum samples at baseline and after 16-week treatment of cholestyramine. Overall, distinguishable shifts in the composition of metabolites were observed before and after treatment (R^2^Y = 0.65, Q^2^Y = 0.165, Figure 4a). Specifically, 14 metabolites were upregulated and 4 were downregulated (median fold change>1.25 or <0.8, *p* < .05, Figure 4b). It was worth noting that several SCFAs were increased in response to the intervention (Figure 4b, 4c). More importantly, patients in two subgroups exhibited differential metabolic alterations (Supplementary SFigure 9). In particular, concentrations of valeric acid and caproic acid were selectively upregulated in the SR group, consistent with the enrichment of SCFA-producing bacteria observed in this group (Figure 4d, e, Supplementary table 3). The metabolic difference between the two subgroups and its consistency with taxonomic changes provides further evidence that the microbial alterations are biologically relevant with the cholestyramine treatment.

### Changes of bile acids, gut microbiome, metabolites, and inflammation were differentially correlated in SR and IR group

**Figure 5.**
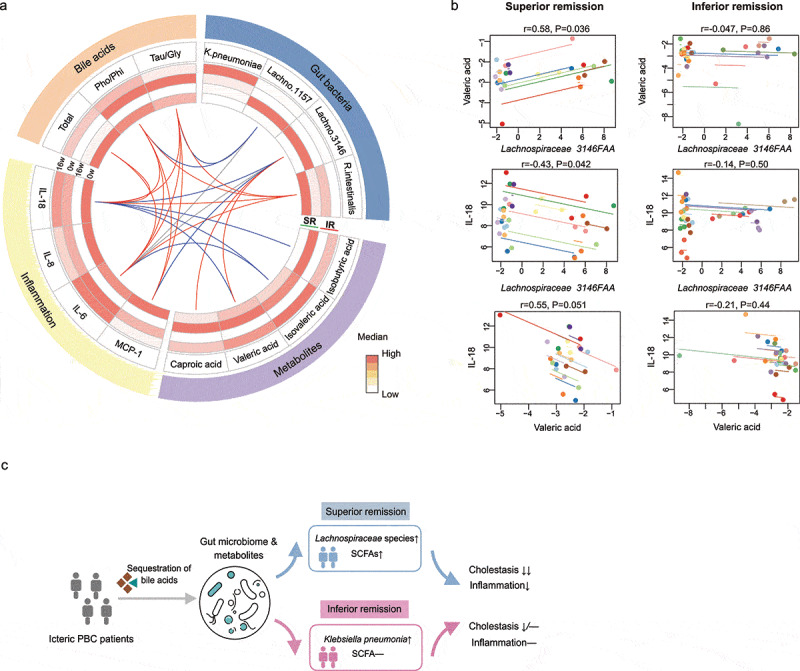
**Divergent correlations among changes of bile acids, gut microbiome, metabolites and inflammation in SR and IR following cholestyramine**. (a) Circos plot showed the correlations between the variations of bile acids, microbiota, metabolites, and inflammatory cytokines in SR group and IR group. Heatmap showed the median levels of the variables in the two subgroups before and after 16-week treatment. Lines indicate significant correlations calculated using rmcorr test (|*r*|>0.3 and *p* < .05). The red and blue edges denote positive and negative correlations in SR, respectively. Grey edges denote significant correlations in IR. (b) Examples of correlations were visualized using rmcorr plot. Each subject’s measurements and the correlation trend line are shown in a different color. The correlation coefficient (positive or negative) is indicated by the direction of the common regression slope. (c) Brief illustration of the study. Changes of gut microbiome and its metabolites may confer the differential responsiveness of PBC patients to the bile acid sequestrants. Tau/Glycine: the ratio of taurine and glycine bile acids; Pho/Phi: the ratio of hydrophobic and hydrophilic bile acids

We next performed a repeated measure correlation analysis (rmcorr) to explore the potential connections of the alterations in bile acids, microbial taxonomy, metabolism and host inflammation following cholestyramine treatment. Multiple associations were identified when analyzing patients in the SR group, while few connections were observed in the IR group. (Figure 5a). In particular, we found strong associations between changes of bile acids and gut bacteria in the SR group (Figure 5a). Furthermore, significant increases of *Lachnospiraceae* species, known for producing SCFAs, correlated with increased levels of valeric acids and caproic acids, as well as reductions of inflammatory markers including IL-18 and MCP-1 in this group (Figure 5b). In addition, SCFAs including valeric acids and isovaleric acids inversely correlated with systemic inflammation only in SR group (Figure 5b).

## Discussion

By modulating endogeneous bile acids through cholestyramine in a cohort of patients with progressive PBC, we identified potential links between bile acids dynamics, compositional, and metabolic changes of gut commensal, and amelioratioin of inflammation and PBC-related cholestasis. A high variability in the remission of cholestasis in PBC following intervention was related to the divergent alterations in microbial taxonomy and metabolites. Of note, subjects with relatively good remission exhibited enrichment of *Lachnospiraceae* species and concordant elevations of SCFAs, which mediated, in part, the anti-cholestatic and anti-inflammatory effects of cholestyramine.

In addition to the decrease of total serum bile acids upon treatment, serum bile acid profiling showed a significant shift toward a more hydrophilic configuration. Hydrophobic bile acids are known to elicit apoptosis and senescence of biliary epithelial cells.^1^ Sequestration of intestinal bile acids downregulated the levels of FGF19; however, serum concentrations of C4, a well-established marker of bile acid synthesis, was not increased accordingly. Herein, we supposed that regulation of bile acid synthesis was more complex in patients with severe liver cholestasis. Despite the downregulation of intestinal FXR/FGF19 by the resin, repression from the intrahepatic FXR/SHP signal remained to be dominated.

Previous work on both rodents and humans consistently suggests that one pharmacological effect of bile acid sequestrant is to augment intestinal activation of the bile acid receptor, TGR5, and thus lead to the release of the downstream hormone GLP-1.^12,23,26–28^ As expected, treatment of cholestyramine induced the release of GLP-1 in our PBC cohort. Mechanistically, although minimal levels of bile acids are capable of reaching the colon, sequestration by a resin would allow an ectopic concentration of bile acids in colon, where TGR5 and GLP-1-producing enteroendocrine L-cells are highly expressed.^26,28^ Moreover, elevation of secondary bile acids in feces resulted in an enhanced activation of TGR5. GLP-1 and its analog extendin-4 can prevent cholangiocyte from apoptosis and facilitate its proliferative repair in response to cholestasis.^24,25^ Therefore, bile acid sequestrant applied in this PBC cohort probably act to augment intestinal TGR5 signaling and colonic production of GLP-1, which then exert cholangioprotective effects.

Impacts of bile acids on the intestinal microbe are complex. The modest alteration at taxonomic level and no differences in the alpha and beta diversity indicated a relatively stable gut microbial community of the samples during the treatment, which was consistent with the results obtained from Mdr2 knockout mice treated with bile acid sequestrants.^12^ Nonetheless, further stratifying the patients according to their clinical responses provided more clues. It is worth noting that the abundance of two species in *the Lachnospiraceae* family were exclusively enriched in the SR group. The *Lachnospiraceae* species are gram-positive and known for producing SCFA, which is generally recognized as one of the most important immunoregulatory molecules metabolized by microbiota.^29^ On the contrary, *Klebsiella pneumoniae*, a gram-negative bacterium with potent lipopolysaccharide, was considered a pro-inflammatory pathobiont and found to cause gut barrier damage and T helper 17 (Th17) cell immune response in PSC.^30^ Our previous study has shown that *Klebsiella penumoniae* was enriched in UDCA-treatment naïve PBC, and correlated positively with the serum level of bilirubin.^8^ Herein, we found that *Klebsiella penumoniae* was selectively elevated in the IR group, which might be responsible for the unfavorable clinical outcomes.

It was suggested that subtle changes in microbial community were able to shift its function profoundly.^31^ As microbial metabolites were regarded as readouts of their function, we further performed targeted metabonomic profiling of microbiota-derived metabolites. Interestingly, SCFAs including valeric acid and caproic acid were increased in SR group. Similar to butyric acid, valeric acids and caproic acids also provide energy for intestinal epithelium and exert anti-inflammtory effects in intestinal and systemic immune diseases.^32–34^ It has been recently reported that valerate provides protection against colitis and multiple sclerosis via promoting Breg differentiation and meanwhile suppressing Th17 cells.^32^ In accordance with our study, it has been reported in animal models of high-fat diet that administration of cholestyramine could increase the fecal and cecal contents of SCFAs.^35–37^ The benefits were further abrogated in germ-free or antibiotic-treated mice, supporting the implication of gut microbiota in the clinical phenotypes conferred by the resin.

With regards to the associations among the variations of microbiota, metabolites, and inflammatory cytokines, substantial differences were found between the two subgroups. SR group possessed a considerably larger number of connections, compared with IR group. Of particular interest was the finding that the enrichment of the *Lachnospiraceae* species was strongly correlated with the increase of circulating valeric acid and caproic acid, as well as the accompanying decrease of inflammatory cytokines, providing further evidence that SCFAs produced by the altered microbiota in the SR group act to mitigate the inflammation and cholestasis.

It remains unclear how cholestyramine-imposed differential impacts on microbital composition and function. The potential factors, including age, sex, BMI, diet and drug use were not biased between SR and IR groups. We next investigated the baseline taxonomy and metabolites and found no single bacteria or metabolite was significantly differed between SR and IR group. Nonetheless, we noticed that the microbial relationships distinguished between the SR and IR groups, which was evidenced by the co-abundance networks of taxa associations. Moreover, the stratified subgroups showed substantially more complex networks than the whole group, implying the microbial ecosystems were virtually not identical among all the subjects. The human intestine harbors a huge number of microbes interacting intricately with each other. Previous studies have suggested that microbial interactions are not only essential for maintaining healthy ecology but also implicated in disease-associated states.^38–42^ In this regard, the differential microbial networks in SR and IR groups at baseline may help explain the variable adaptation to the bile acid fluctuations. Therefore, in addition to the microbial composition, it is critical to characterize the interactions between the microbes and determine the underlying mechanisms.

There are several limitations in the study. First, cholestyramine is a traditional bile acid sequestrant with a taste intolerant for part of patients. However, second generation of bile acid sequestrants are currently unavailable in China and cholestyramine was thus employed to sequestrate intestinal bile acids in an effort to investigate the bile acid-microbiota crosstalk. Second, the study is of relatively modest sample size. Nonetheless, PBC is not a common disease and the UDCA treatment is effective in more than half of patients. Third, the findings in this work are correlational as the differential responses of the human gut microbiome to cholestyramine provide a potential explanation for the clinical effects observed. Future studies are needed to elucidate the underlying causality.

## Conclusion

In conclusion, we provide a unique perspective into the dynamic changes of the gut microbiome in response to bile acid modulation of PBC-related cholestasis. Patients administrated cholestyramine demonstrated heterogeneous but overall advantageous responses, which were largely mediated by gut commensal. Given the suboptimal therapeutic strategies for progressive PBC, this real-world study highlighted the possibilities for implementation of microbiota and its metabolite-targeted treatment in the future.

## Supplementary Material

Supplemental Material

## Data Availability

Data are available upon request from the corresponding author Dr. Xiong Ma.

## List of Abbreviations

ALP, alkaline phosphatase; ALT, alanine aminotransferase; AST, aspartate aminotransferase; ALB, albumin; BMI, Body mass index; C4, 7α-hydroxy-4-cholesten-3-one; CA: cholic acid; CHO, cholesterol; CYP7A1, cholesterol 7α-hydroxylase; CDCA: chenodeoxycholic acid; Cr: creatinine; DCA: deoxycholic acid; FGF19, fibroblast growth factor 19; GGT, γ-glutamyl transpeptidase; GLP-1, glucagon-like peptide-1; GUDCA: glycoursodeoxycholic acid; GCA: glycocholic acid; GCDCA: glycochenodeoxycholate; GDCA: glycodeoxycholic acid; GHCA: glycohyocholate; GLCA: glycolithocholate; HDCA: α-hyodeoxycholic acid; HCA: γ-muricholic acid\hyocholic acid; IgM, immunoglobulin M; IgG, immunoglobulin G; IgA, immunoglobulin A; INR: international normalized ratio; LCA: lithocholic acid; MCP-1, monocyte chemoattractant protein-1; PBC: primary biliary cholangitis; Pho/Phi: the ratio of hydrophobic and hydrophilic bile acids; SCFAs: short-chain fatty acids; TNF-α, Tumor Necrosis Factor-α; Tau/Glycine: the ration of taurine and glycine bile acids; TCA: taurocholic acid; TCDCA: taurochenodeoxycholate; TUDCA: tauroursodeoxycholic acid; THCA: taurohyocholate; TDCA: taurodeoxycholate; TLCA: taurolithocholate; UPLC-MS/MS: ultra-performance liquid chromatography coupled to tandem mass spectrometry; UDCA: ursodeoxycholic acid.

## References

[1] Terziroli Beretta-Piccoli B, Mieli-Vergani G, Vergani D, Vierling JM, Adams D, Alpini G, Banales JM, Beuers U, Björnsson E, Bowlus C, et al. The challenges of primary biliary cholangitis: what is new and what needs to be done[J]. J Autoimmun. 2019; 105:102328. 31548157 10.1016/j.jaut.2019.102328

[2] Clinical Practice EASL. Guidelines: the diagnosis and management of patients with primary biliary cholangitis[J]. J Hepatol. 2017;67:145–15. 28427765 10.1016/j.jhep.2017.03.022

[3] Wagner M, Fickert P. Drug therapies for chronic cholestatic liver diseases. Annu Rev Pharmacol Toxicol. 2020;60:503–527. doi:10.1146/annurev-pharmtox-010818-021059. 31506007

[4] Joyce SA, Gahan CG. Bile acid modifications at the microbe-host interface: potential for nutraceutical and pharmaceutical interventions in host health[J]. Annu Rev Food Sci Technol. 2016;7(1):313–333. doi:10.1146/annurev-food-041715-033159. 26772409

[5] Chen ML, Takeda K, Sundrud MS. Emerging roles of bile acids in mucosal immunity and inflammation[J]. Mucosal Immunol. 2019;12(4):851–861. doi:10.1038/s41385-019-0162-4. 30952999

[6] Liu Y, Chen K, Li F, Gu Z, Liu Q, He L, Shao T, Song Q, Zhu F, Zhang L, et al. Probiotic Lactobacillus rhamnosus GG Prevents Liver Fibrosis Through Inhibiting Hepatic Bile Acid Synthesis and Enhancing Bile Acid Excretion in Mice. Hepatology. 2020;71(6):2050–2066. doi:10.1002/hep.30975. 31571251 PMC7317518

[7] Friedman ES, Li Y, Shen TCD, Jiang J, Chau L, Adorini L, Babakhani F, Edwards J, Shapiro D, Zhao C, et al. FXR-Dependent Modulation of the Human Small Intestinal Microbiome by the Bile Acid Derivative Obeticholic Acid[J]. Gastroenterology. 2018;155(6):1741–1752. e1745.. doi:10.1053/j.gastro.2018.08.022. 30144429 PMC6279623

[8] Tang R, Wei Y, Li Y, Chen W, Chen H, Wang Q, Yang F, Miao Q, Xiao X, Zhang H, et al. Gut microbial profile is altered in primary biliary cholangitis and partially restored after UDCA therapy[J]. Gut. 2018;67(3):534–541. doi:10.1136/gutjnl-2016-313332. 28213609

[9] Li B, Selmi C, Tang R, Gershwin ME, Ma X. The microbiome and autoimmunity: a paradigm from the gut–liver axis. Cell Mol Immunol. 2018;15(6):595–609. doi:10.1038/cmi.2018.7. 29706647 PMC6079090

[10] Chen W, Wei Y, Xiong A, Li Y, Guan H, Wang Q, Miao Q, Bian Z, Xiao X, Lian M, et al. Comprehensive Analysis of Serum and Fecal Bile Acid Profiles and Interaction with Gut Microbiota in Primary Biliary Cholangitis[J]. Clin Rev Allergy Immunol. 2020. 58(1): 25-38. 10.1007/s12016-019-08731-2 30900136

[11] Sjoberg BG, Straniero S, Angelin B, Rudling M. Cholestyramine treatment of healthy humans rapidly induces transient hypertriglyceridemia when treatment is initiated[J]. Am J Physiol Endocrinol Metab. 2017;313(2):E167–e174. doi:10.1152/ajpendo.00416.2016. 28487440

[12] Fuchs CD, Paumgartner G, Mlitz V, Kunczer V, Halilbasic E, Leditznig N, Wahlström A, Ståhlman M, Thüringer A, Kashofer K, et al. Colesevelam attenuates cholestatic liver and bile duct injury in Mdr2−/− mice by modulating composition, signalling and excretion of faecal bile acids. Gut. 2018;67(9):1683–1691. doi:10.1136/gutjnl-2017-314553. 29636383 PMC6109278

[13] Polter DE, Gruhl V, Eigenbrodt EH, Combes B. Beneficial effect of cholestyramine in sclerosing cholangitis[J]. Gastroenterology. 1980;79(2):326–333. doi:10.1016/0016-5085(80)90149-3. 7399237

[14] Xie G, Wang Y, Wang X, Zhao A, Chen T, Ni Y, Wong L, Zhang H, Zhang J, Liu C, et al. Profiling of Serum Bile Acids in a Healthy Chinese Population Using UPLC–MS/MS. J Proteome Res. 2015;14(2):850–859. doi:10.1021/pr500920q. 25581415

[15] Zhu P, Zhang J, Chen Y, Yin S, Su M, Xie G, Brouwer KLR, Liu C, Lan K, Jia W, et al. Analysis of human C24 bile acids metabolome in serum and urine based on enzyme digestion of conjugated bile acids and LC-MS determination of unconjugated bile acids[J]. Anal Bioanal Chem. 2018;410(21):5287–5300. doi:10.1007/s00216-018-1183-7. 29907951 PMC6424582

[16] Zhao L, Ni Y, Su M, Li H, Dong F, Chen W, Wei R, Zhang L, Guiraud SP, Martin F-P, et al. High Throughput and Quantitative Measurement of Microbial Metabolome by Gas Chromatography/Mass Spectrometry Using Automated Alkyl Chloroformate Derivatization[J]. Anal Chem. 2017;89(10):5565–5577. doi:10.1021/acs.analchem.7b00660. 28437060 PMC5663283

[17] Segata N, Waldron L, Ballarini A, Narasimhan V, Jousson O, Huttenhower C. Metagenomic microbial community profiling using unique clade-specific marker genes[J]. Nat Methods. 2012;9(8):811–814. doi:10.1038/nmeth.2066. 22688413 PMC3443552

[18] Franzosa EA, McIver LJ, Rahnavard G, Thompson LR, Schirmer M, Weingart G, Lipson KS, Knight R, Caporaso JG, Segata N, et al. Species-level functional profiling of metagenomes and metatranscriptomes[J]. Nat Methods. 2018;15(11):962–968. doi:10.1038/s41592-018-0176-y. 30377376 PMC6235447

[19] Friedman J, Alm EJ, von Mering C. Inferring correlation networks from genomic survey data[J]. PLoS Comput Biol. 2012;8(9):e1002687. doi:10.1371/journal.pcbi.1002687. 23028285 PMC3447976

[20] Shannon P, Markiel A, Ozier O, Baliga NS, Wang JT, Ramage D, Amin N, Schwikowski B, Ideker T. Cytoscape: a software environment for integrated models of biomolecular interaction networks[J]. Genome Res. 2003;13(11):2498–2504. doi:10.1101/gr.1239303. 14597658 PMC403769

[21] Pinto C, Giordano DM, Maroni L, Marzioni M. Role of inflammation and proinflammatory cytokines in cholangiocyte pathophysiology[J]. Biochim Biophys Acta Mol Basis Dis. 2018;1864(4):1270–1278. doi:10.1016/j.bbadis.2017.07.024. 28754451

[22] Heuman DM. Quantitative estimation of the hydrophilic-hydrophobic balance of mixed bile salt solutions. J Lipid Res. 1989;30(5):719–730. doi:10.1016/S0022-2275(20)38331-0. 2760545

[23] Thomas C, Gioiello A, Noriega L, Strehle A, Oury J, Rizzo G, Macchiarulo A, Yamamoto H, Mataki C, Pruzanski M, et al. TGR5-mediated bile acid sensing controls glucose homeostasis[J]. Cell Metab. 2009;10(3):167–177. doi:10.1016/j.cmet.2009.08.001. 19723493 PMC2739652

[24] Marzioni M, Alpini G, Saccomanno S, Candelaresi C, Venter J, Rychlicki C, Fava G, Francis H, Trozzi L, Glaser S, et al. Glucagon-like peptide-1 and its receptor agonist exendin-4 modulate cholangiocyte adaptive response to cholestasis[J]. Gastroenterology. 2007;133(1):244–255. doi:10.1053/j.gastro.2007.04.007. 17631146

[25] Marzioni M, Alpini G, Saccomanno S, Candelaresi C, Venter J, Rychlicki C, Fava G, Francis H, Trozzi L, Benedetti A, et al. Exendin-4, a glucagon-like peptide 1 receptor agonist, protects cholangiocytes from apoptosis[J]. Gut. 2009;58(7):990–997. doi:10.1136/gut.2008.150870. 18829977 PMC2695839

[26] Harach T, Pols TW, Nomura M, Maida A, Watanabe M, Auwerx J, Schoonjans K. TGR5 potentiates GLP-1 secretion in response to anionic exchange resins[J]. Sci Rep. 2012;2(1):430. doi:10.1038/srep00430. 22666533 PMC3362799

[27] Beysen C, Murphy EJ, Deines K, Chan M, Tsang E, Glass A, Turner SM, Protasio J, Riiff T, Hellerstein MK, et al. Effect of bile acid sequestrants on glucose metabolism, hepatic de novo lipogenesis, and cholesterol and bile acid kinetics in type 2 diabetes: a randomised controlled study[J]. Diabetologia. 2012;55(2):432–442. doi:10.1007/s00125-011-2382-3. 22134839

[28] Potthoff MJ, Potts A, He T, Duarte JAG, Taussig R, Mangelsdorf DJ, Kliewer SA, Burgess SC. Colesevelam suppresses hepatic glycogenolysis by TGR5-mediated induction of GLP-1 action in DIO mice[J]. Am J Physiol Gastrointest Liver Physiol. 2013;304(4):G371–380. doi:10.1152/ajpgi.00400.2012. 23257920 PMC3566618

[29] Postler TS, Ghosh S. Understanding the Holobiont: how Microbial Metabolites Affect Human Health and Shape the Immune System[J]. Cell Metab. 2017;26(1):110–130. doi:10.1016/j.cmet.2017.05.008. 28625867 PMC5535818

[30] Nakamoto N, Sasaki N, Aoki R, Miyamoto K, Suda W, Teratani T, Suzuki T, Koda Y, Chu P-S, Taniki N, et al. Gut pathobionts underlie intestinal barrier dysfunction and liver T helper 17 cell immune response in primary sclerosing cholangitis[J]. Nat Microbiol. 2019;4(3):492–503. doi:10.1038/s41564-018-0333-1. 30643240

[31] Liu Y, Wang Y, Ni Y, Cheung CKY, Lam KSL, Wang Y, Xia Z, Ye D, Guo J, Tse MA, et al. Gut Microbiome Fermentation Determines the Efficacy of Exercise for Diabetes Prevention[J]. Cell Metab. 2020;31(1):77–91- e75. doi:10.1016/j.cmet.2019.11.001. 31786155

[32] Luu M, Pautz S, Kohl V, Singh R, Romero R, Lucas S, Hofmann J, Raifer H, Vachharajani N, Carrascosa LC, et al. The short-chain fatty acid pentanoate suppresses autoimmunity by modulating the metabolic-epigenetic crosstalk in lymphocytes[J]. Nat Commun. 2019;10(1):760. doi:10.1038/s41467-019-08711-2. 30770822 PMC6377655

[33] De Preter V, Machiels K, Joossens M, Arijs I, Matthys C, Vermeire S, Rutgeerts P, Verbeke K. Faecal metabolite profiling identifies medium-chain fatty acids as discriminating compounds in IBD[J]. Gut. 2015;64(3):447–458. doi:10.1136/gutjnl-2013-306423. 24811995

[34] Jørgensen JR, Clausen MR, Mortensen PB. Oxidation of short and medium chain C2-C8 fatty acids in Sprague-Dawley rat colonocytes. Gut. 1997;40(3):400–405. doi:10.1136/gut.40.3.400. 9135532 PMC1027093

[35] Kusumoto Y, Irie J, Iwabu K, Tagawa H, Itoh A, Kato M, Kobayashi N, Tanaka K, Kikuchi R, Fujita M, et al. Bile acid binding resin prevents fat accumulation through intestinal microbiota in high-fat diet-induced obesity in mice[J]. Metabolism. 2017;71:1–6. doi:10.1016/j.metabol.2017.02.011. 28521862

[36] Alexander C, Guard BC, Suchodolski JS, Swanson KS. Cholestyramine decreases apparent total tract macronutrient digestibility and alters fecal characteristics and metabolites of healthy adult dogs1. J Anim Sci. 2019;97(3):1020–1026. doi:10.1093/jas/sky437. 30423121 PMC6396232

[37] Nishida S, Horinouchi A, Higashimura Y, Akahori R, Matsumoto K. Cholestyramine, a Bile Acid Sequestrant, Increases Cecal Short Chain Fatty Acids and Intestinal Immunoglobulin A in Mice[J]. Biol Pharm Bull. 2020;43(3):565–568. doi:10.1248/bpb.b19-00923. 31852854

[38] Coyte KZ, Rakoff-Nahoum R-NS. Understanding Competition and Cooperation within the Mammalian Gut Microbiome. Curr Biol. 2019;29(11):R538–r544. doi:10.1016/j.cub.2019.04.017. 31163167 PMC6935513

[39] Chen L, Collij V, Jaeger M, van den Munckhof ICL, Vich Vila A, Kurilshikov A, Gacesa R, Sinha T, Oosting M, Joosten LAB, et al. Gut microbial co-abundance networks show specificity in inflammatory bowel disease and obesity[J]. Nat Commun. 2020;11(1):4018. doi:10.1038/s41467-020-17840-y. 32782301 PMC7419557

[40] Faust K, Sathirapongsasuti JF, Izard J, Segata N, Gevers D, Raes J, Huttenhower C. Microbial co-occurrence relationships in the human microbiome[J]. PLoS Comput Biol. 2012;8(7):e1002606. doi:10.1371/journal.pcbi.1002606. 22807668 PMC3395616

[41] Yilmaz B, Juillerat P, Øyås O, Ramon C, Bravo FD, Franc Y, Fournier N, Michetti P, Mueller C, Geuking M, et al. Microbial network disturbances in relapsing refractory Crohn’s disease[J]. Nat Med. 2019;25(2):323–336. doi:10.1038/s41591-018-0308-z. 30664783

[42] Wang J, Zheng J, Shi W, Du N, Xu X, Zhang Y, Ji P, Zhang F, Jia Z, Wang Y, et al. Dysbiosis of maternal and neonatal microbiota associated with gestational diabetes mellitus[J]. Gut. 2018;67(9):1614–1625. doi:10.1136/gutjnl-2018-315988. 29760169 PMC6109274

