# Supplementary material for: Alterations in microbiota and their metabolites are associated with beneficial effects of bile acid sequestrant on icteric primary biliary Cholangitis
Source: Gut Microbes. 2021 Aug 26;13(1):1946366. doi: 10.1080/19490976.2021.1946366 (PMC8405155; doi:10.1080/19490976.2021.1946366)

Supplementary figure 1. (A) The ratio of taurine- and glycine-conjugated bile acid decreased following cholestyramine treatment. (B) Separate analysis of taurine- and glycine-conjugated bile acid within SR and IR group. (C) Serum levels of C4 were measured by UPLC-MS/MS before and after 16-week intervention of cholestyramine. A paired Wilcoxon rank sum test was used. *p<0.05, ** p<0.01, **** p<0.0001.

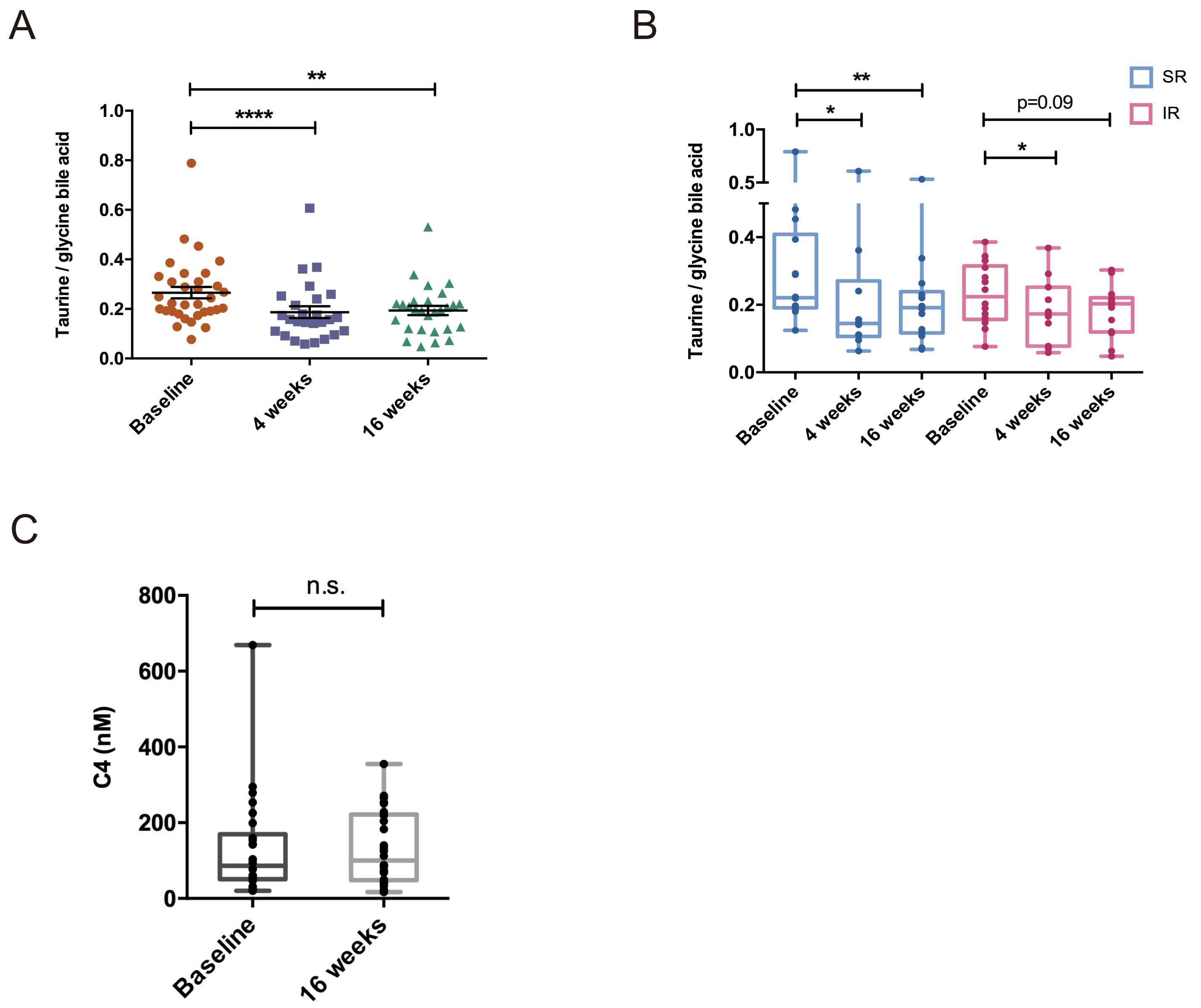

Supplementary figure 2. Effects of 16-week treatment of cholestyramine on fecal bile acid profile. (A) Dot plots (with mean±SEM) showed the dynamics of 26 fecal bile acids at 0 and 16 weeks of cholestyramine treatment in all subjects; (B) Compositional shift of fecal bile acids at baseline and 16 weeks; Changes of fecal (C) total bile acids, (D) secondary bile acids, (E) primary bile acids, (F) unconjugated bile acids, (G) conjugated bile aicds and (H) Heuman index of fecal bile acids; Separate analysis of (I) secondary bile acids and (J) unconjugated bile acids within group SR and IR. A paired Wilcoxon rank sum test was used. * p<0.05.

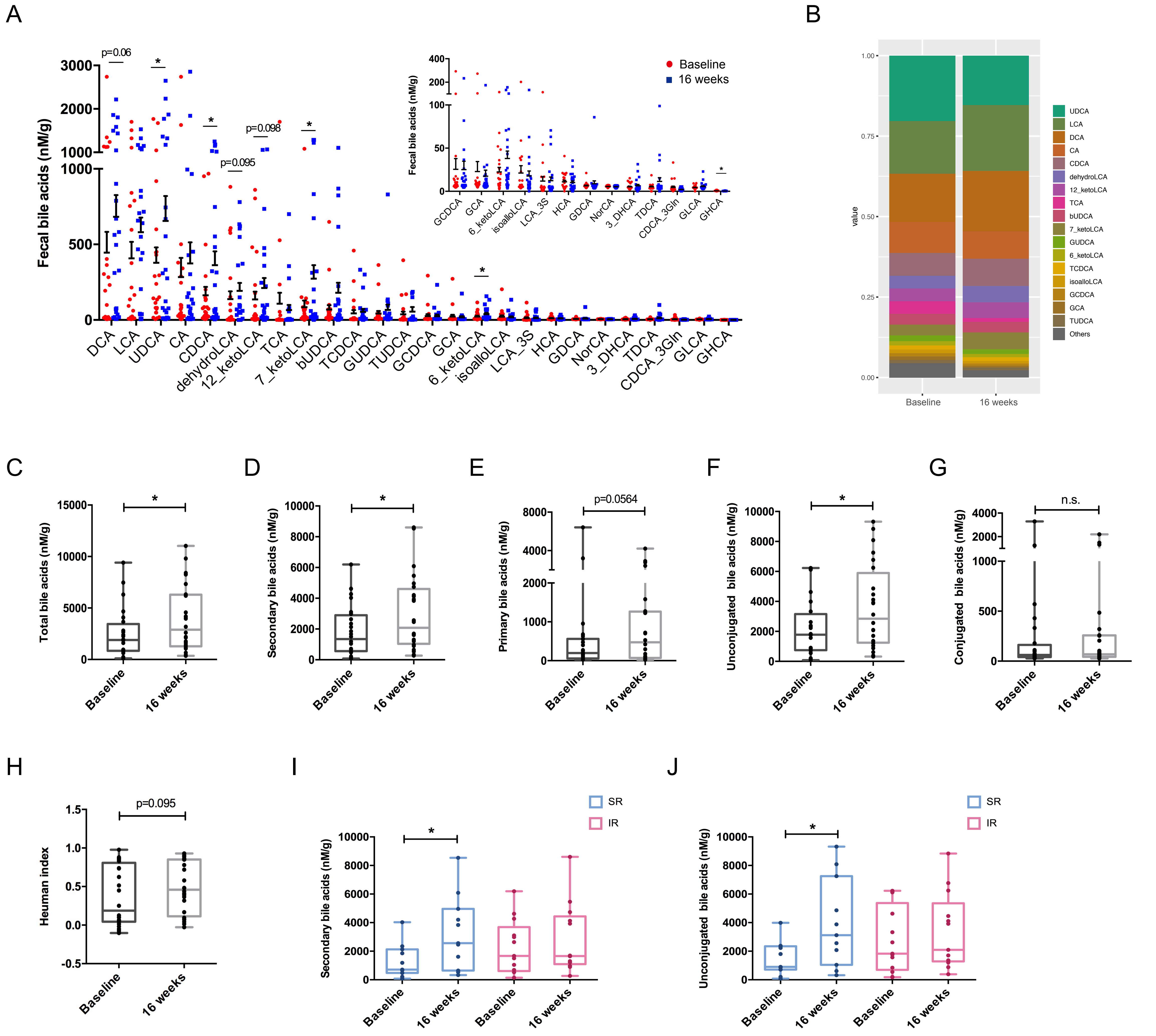

Supplementary Figure 3. The relative abundance of the top 30 most abundant species from all samples grouped by treatment.

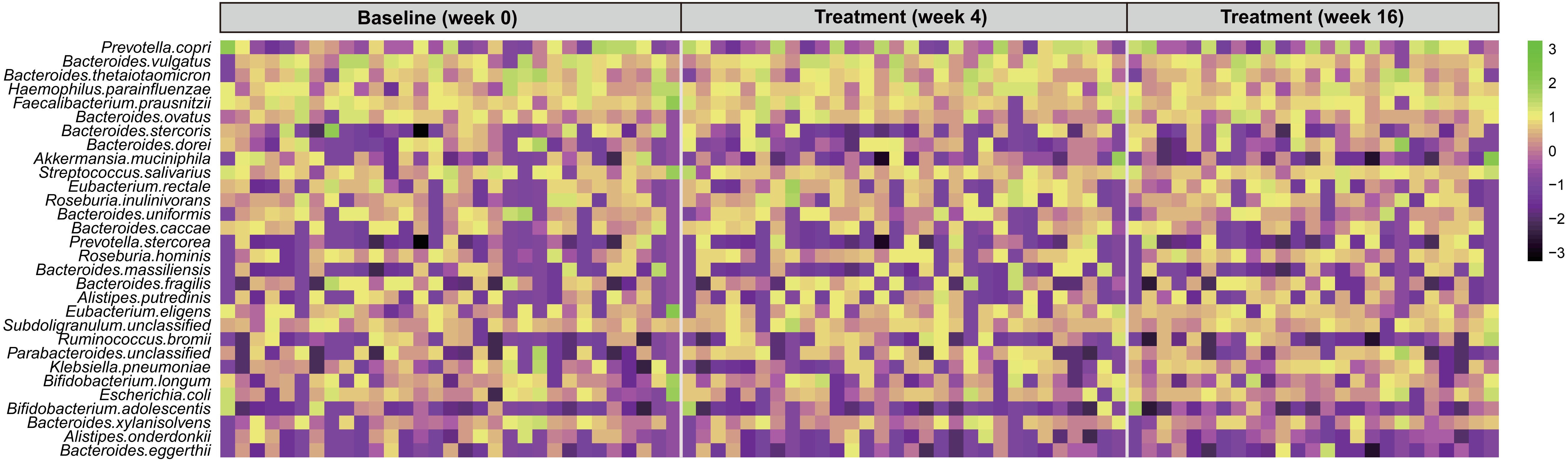

Supplementary Figure 4. Associations of changes in species and clinical improvement during cholestyramine treatment. Repeated measure correlation (rmcorr) method was used to calculate the correlation coeffcient. *denotes<0.05; ** denotes p<0.01.

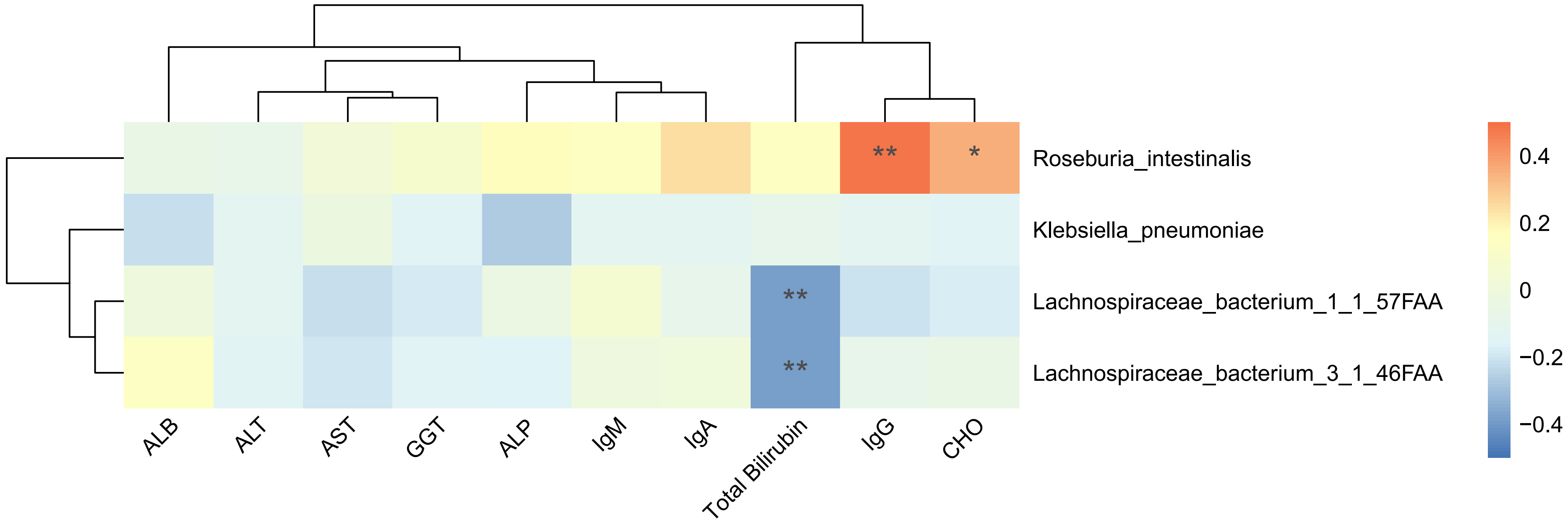

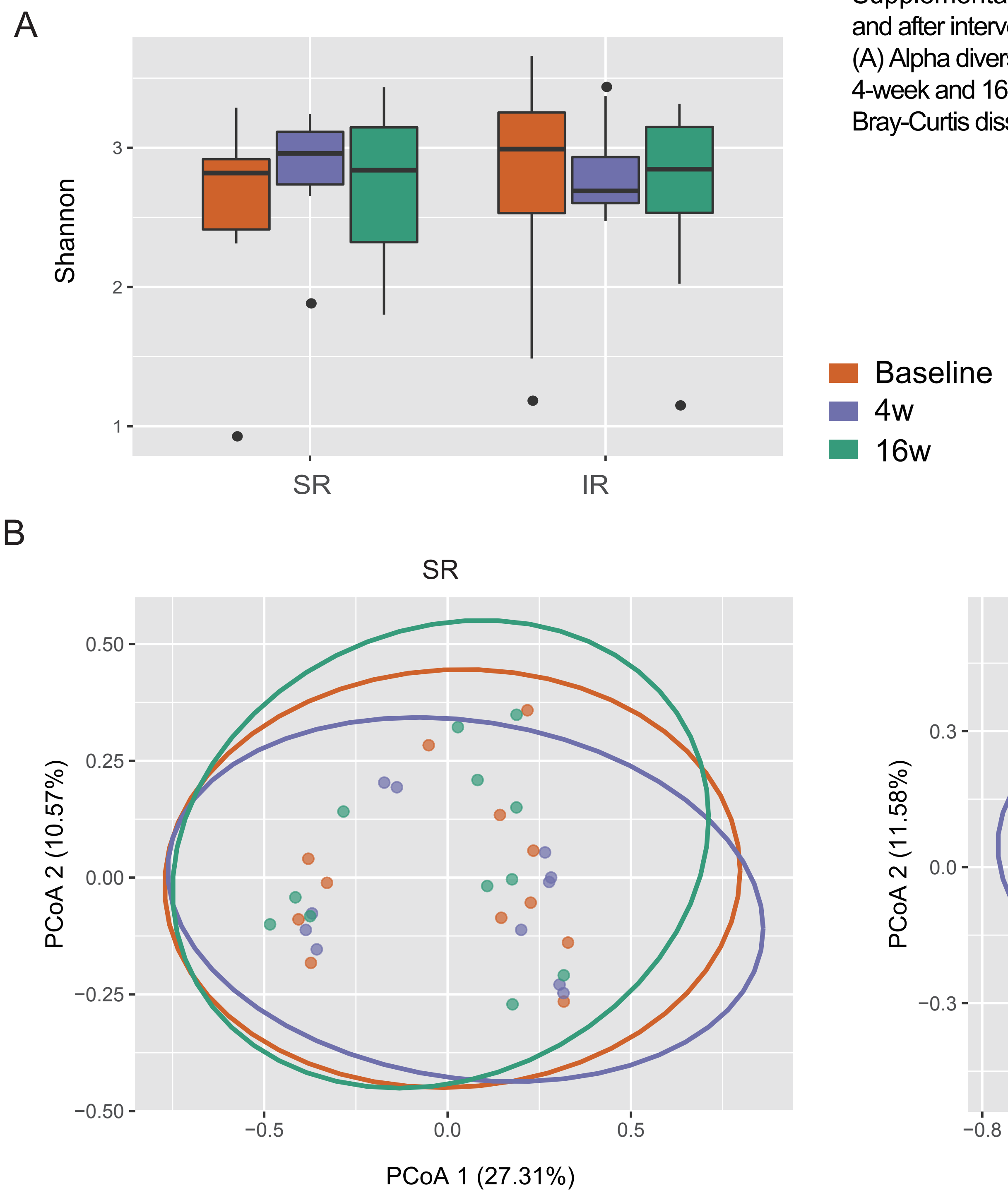

Supplementary Figure 5. No significant difference in alpha or beta diversity before and after intervention was found in subjects with superior remission or inferior remission. (A) Alpha diversity (Shannon index) in SR and IR was measured at baseline and after 4-week and 16-week treatment. (B) Principal coordinate analysis (PCoA) based on Bray-Curtis dissimilarities in SR and IR was performed in response to treatment.

Supplementary figure 6. Associations of changes in fecal bile acids and species in (A) group SR and (B) IR during cholestryramine treatment. Repeated measure correlation (rmcorr) method was used to calculate the correlation coeffcient. *denotes p<0.05, **denotes p<0.01.Con: conjugated bile acids; hydro index: hydrophobicity index of total bile acids; uncon: unconjugated bile acids; TSBA: total secondary bile acids; sum: sum bile acids; TS.P: total secondary/primary bile acid; uncon.con: unconjugated/conjugated bile acid.

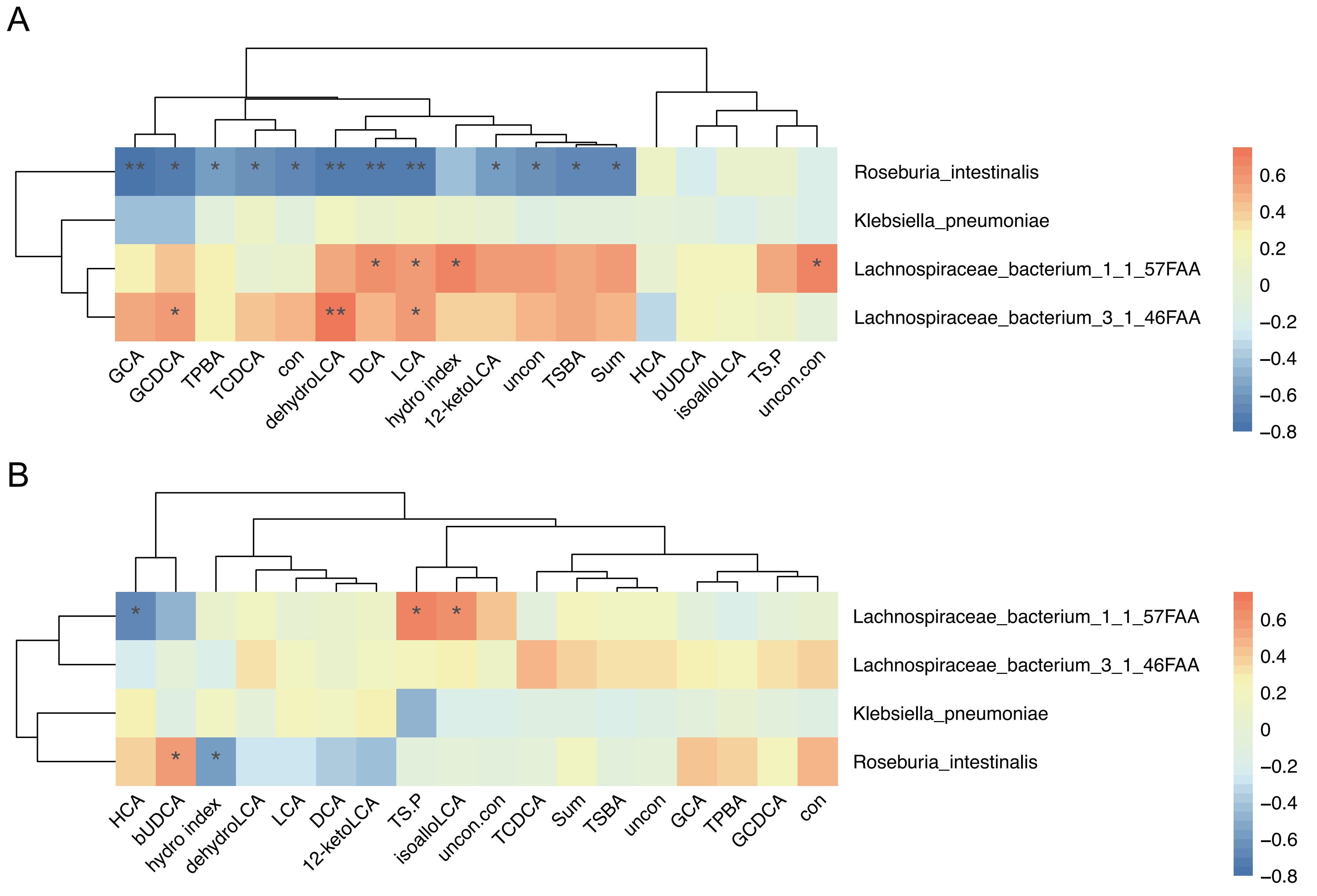

Supplementary Figure 7. Microbial correlations at species level of all samples at baseline (A) and after 16-week treatment (B) were visualized in Cytoscape. Strong correlations (|r| value > 0.53 and P < 0.05) are depicted. The red and blue edges denote positive and negative correlations, respectively. The color of the nodes is based on phylum and the size is based on edges connected to the nodes.

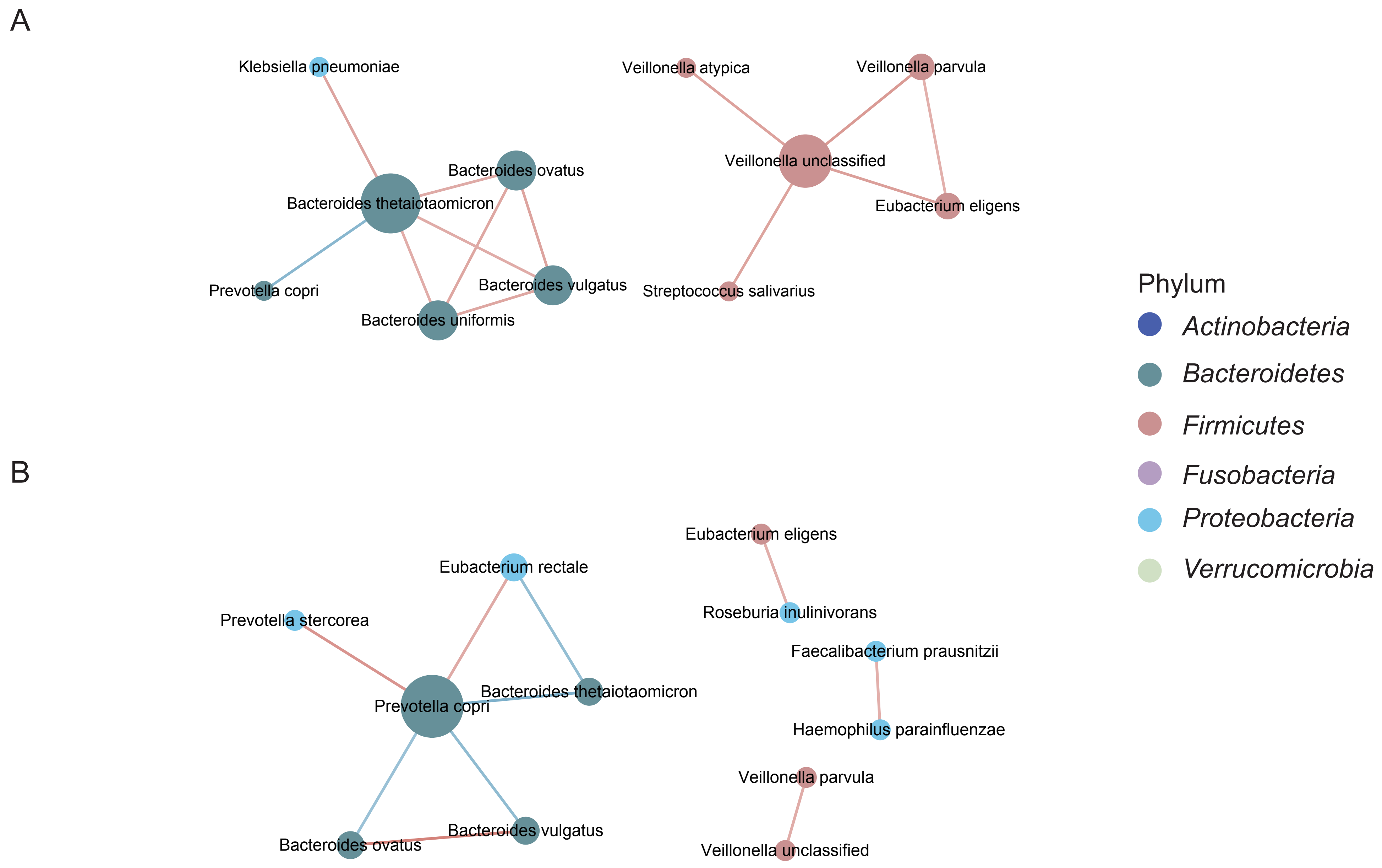

Supplementary Figure 8. Key species identified by co-abundance network analysis showed different alterations in response to treatment between SR and IR. *p<0.05, ** p< 0.01 .

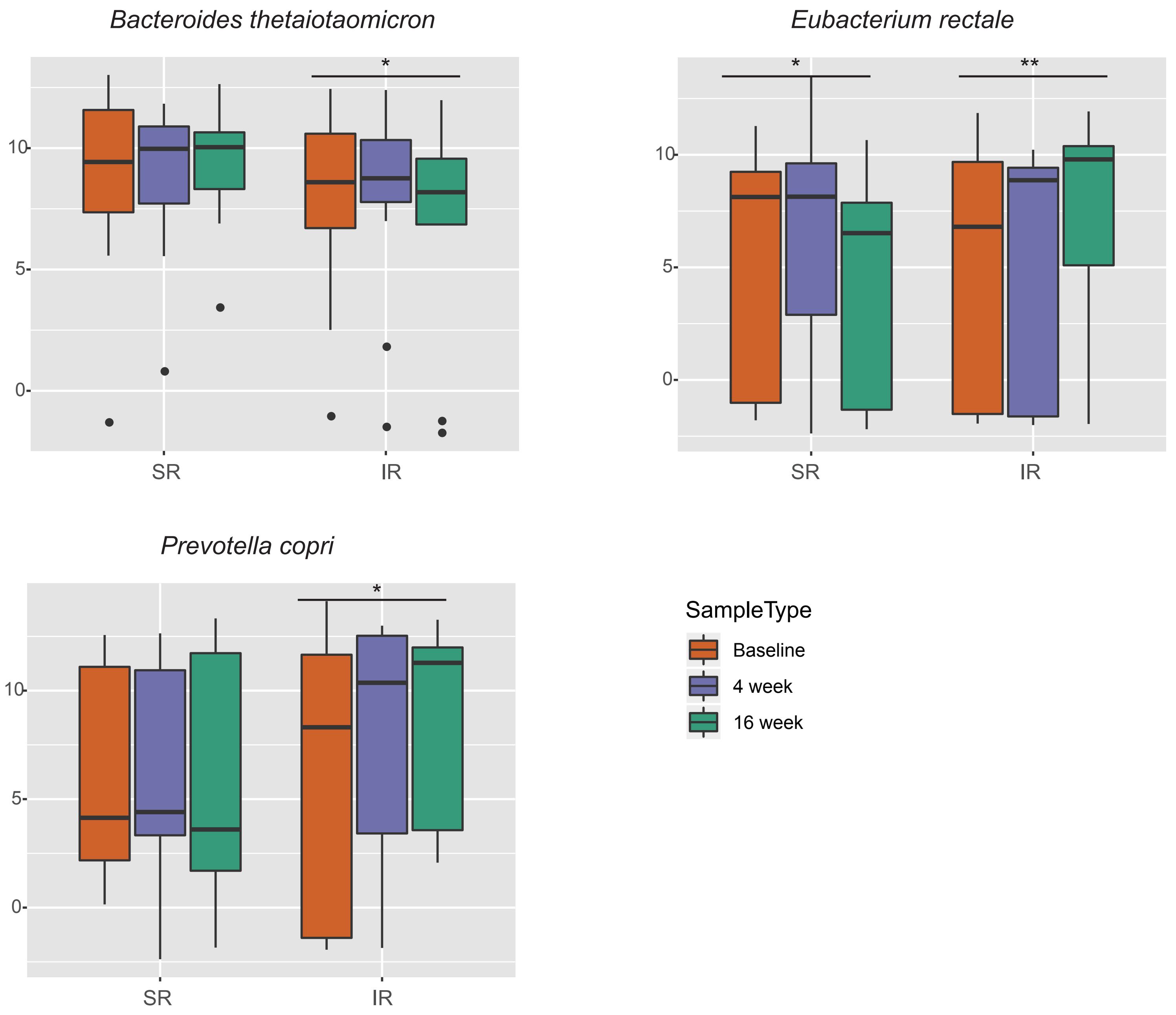

Supplementary Figure 9. Volcano plots highlighed the metabolites that changed after 16-week treatment in group with superior remission (A) and group with inferior remission (B). The y-axis corresponds to log 10 P value, and the x-axis represents log2 median fold change in each group. Fold change was defined as the ratio of concentration of the metabolites after 16-week treatment to that at baseline. Red and blue dots denote increased and decreased metabolites, respectively.

A

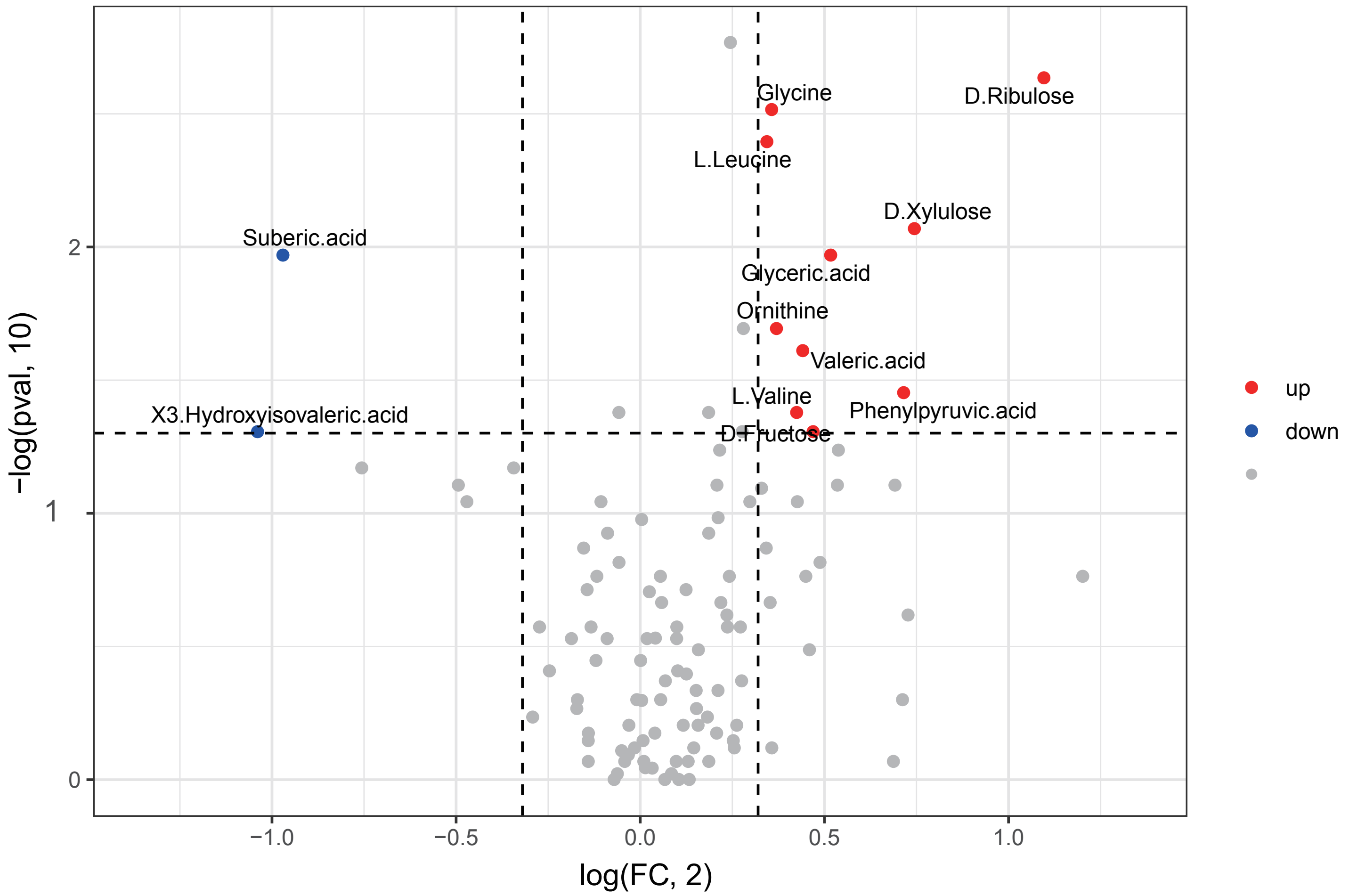

B

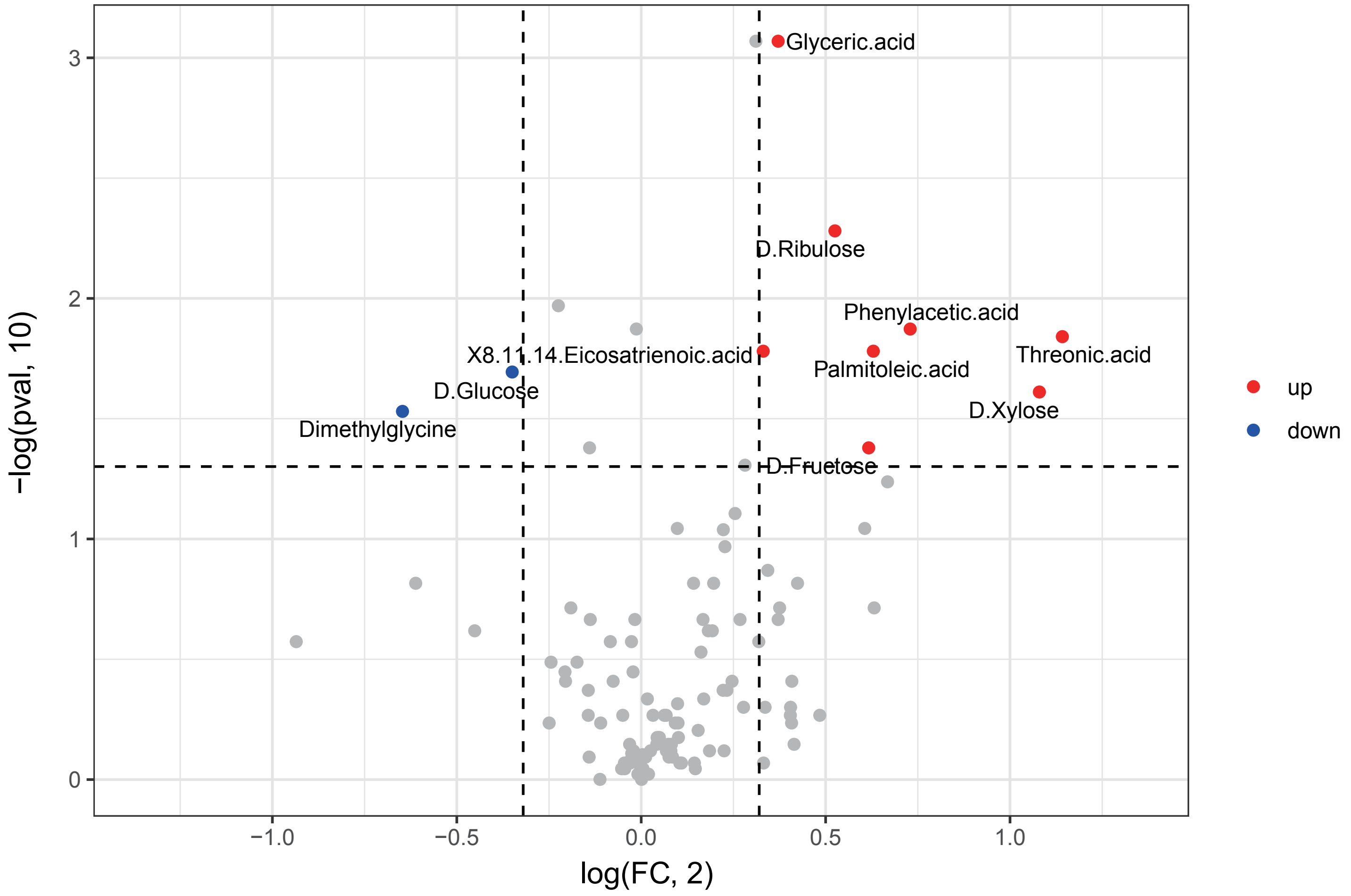

Supplement: Supplemental Material [file KGMI_A_1946366_SM3457.zip › supplementary/downloadFromZipFile.pdf]
